# New Molecules in Type 2 Diabetes: Advancements, Challenges and Future Directions

**DOI:** 10.3390/ijms25116218

**Published:** 2024-06-05

**Authors:** Kyriazoula Chatzianagnostou, Melania Gaggini, Adrian Suman Florentin, Ludovica Simonini, Cristina Vassalle

**Affiliations:** 1 Fondazione CNR-Regione Toscana G Monasterio, Via G. Moruzzi 1, 56124 Pisa, Italy; zulachat@ftgm.it; 2 Institute of Clinical Physiology, National Research Council, Via G. Moruzzi 1, 56124 Pisa, Italy; melania.gaggini@cnr.it (M.G.); adriansumanflorentin@gmail.com (A.S.F.); 3 Department of Surgical, Medical and Molecular Pathology and Critical Area, University of Pisa, 56126 Pisa, Italy; simoniniludovica@gmail.com

**Keywords:** cardiometabolic conditions, atherosclerosis, type 2 diabetes mellitus, GLP-1 receptor agonists, SGLT2 inhibitors

## Abstract

Although good glycemic control in patients with type 2 diabetes (T2D) can prevent cardiovascular complications, many diabetic patients still have poor optimal control. A new class of antidiabetic drugs (e.g., glucagon-like peptide-1-GLP-1 receptor agonists, sodium-glucose co-transporters-SGLT2 inhibitors), in addition to the low hypoglycemic effect, exert multiple beneficial effects at a metabolic and cardiovascular level, through mechanisms other than antihyperglycemic agents. This review aims to discuss the effects of these new antidiabetic drugs, highlighting cardiovascular and metabolic benefits, through the description of their action mechanisms as well as available data by preclinical and clinical studies. Moreover, new innovative tools in the T2D field will be described which may help to advance towards a better targeted T2D personalized care in future.

## 1. Introduction

Diabetes is a global pandemic; a significant portion of the world’s population is currently affected by diabetes, with current trends suggesting that these rates will continue to rise [1]. Specifically, the worldwide prevalence of this disease in 20–79-year-old people (similar in the two sexes, and highest in elderly subjects) in 2021 was estimated to be 10.5% (536.6 million people), increasing to 12.2% (783.2 million) in 2045 [2]. Type 2 diabetes (T2D) patients also show a high cardiovascular risk, as a large percentage of these patients frequently experience cardiovascular events [3,4,5]. In fact, the status of chronic hyperglycemia, visceral adiposity, hypertension and increased arterial stiffness leads to the increased risk of cardiovascular disease in diabetic patients who have higher mortality and morbidity than the rest of the population; it has been estimated that more than 70% of T2D patients die of cardiovascular causes [6]. What is worrying is that, although good glycemic control in patients with T2D can prevent cardiovascular complications, many diabetic patients still have poor optimal glycemic control. In this context, a new class of antidiabetic drugs (e.g., GLP-1 receptor agonists, SGLT2 inhibitors), in addition to the hypoglycemic effect, exert multiple beneficial actions at the metabolic and cardiovascular level through mechanisms other than glycemic control [7]. Thus, T2D care is evolving beyond glycemic control, which was the focus of treatment for a long time, being now increasingly based on cardiovascular risk stratification.

This review aims to discuss action mechanisms and available preclinical and clinical studies of these new antidiabetic drugs, beyond their role as oral antihyperglycemic agents in T2D, highlighting cardiovascular and metabolic benefits. Moreover, future research directions on innovative therapeutic tools will be reported, which may help to advance future clinical management of T2D and cardiometabolic diseases towards personalized treatments and minimizing adverse effects.

## 2. GLP-1 Receptor Agonists

The “ominous octet” term refers to the various parameters that impact the pathophysiology of T2D, including a decreased insulin secretion by pancreatic β-cells and an increased glucagon secretion by pancreatic α-cells, an increased hepatic glucose production, an increased lipolysis, an impaired brain appetite regulation, the decreased incretin effect at the intestinal level, increased renal glucose reabsorption and decrease in muscle glucose uptake, which represent the core to whom sharpen the T2D treatment (Table 1) [8]. In this context, glucagon-like peptide-1 agents (GLP-1) are of particular importance; these molecules belong to the family of incretin hormones (gastrointestinal hormones released after nutrient intake with the ability to glucose-dependently augment insulin secretory responses during periods characterized by hyperglycemia), and exert their action through GLP-1R, a G-protein coupled receptor.

Following the meal, GLP-1 is released by intestinal cells but it is rapidly degraded by a specific enzyme, dipeptidyl peptidase 4 (DPP-4). The release of GLP-1 is reduced progressively going from normoglycemia, to preT2D and finally to T2D; for this reason, its use for therapeutic purposes is not practicable, except with a continuous infusion. To overcome the problem of the rapid degradation of GLP-1, analogues have been developed, more correctly defined as GLP-1 receptor agonists (GLP-1 RAs), with a structure more or less similar to GLP-1, which resist the degradation exerted by DPP-4 and which are sometimes linked to molecules that slow down their subcutaneous absorption (Table 2). These pharmacotherapies have been designed through modification of either native human GLP-1 (liraglutide, semaglutide, dulaglutide and albiglutide) or exendin-4 GLP-1 peptides (exenatide and lixisenatide) [9].

GLP-1 RAs are administered by subcutaneous injection (like insulin) once a day (liraglutide, lixisenatide) or twice a day (exenatide) or once a week (long-acting exenatide, dulaglutide, subcutaneous semaglutide). Liraglutide and lixisenatide are also available in pre-constituted combinations with insulin (degludec and glargine, respectively) in fixed proportions. Recently, an oral GLP-1 drug is being developed, represented by oral semaglutide, an innovative oral drug for the treatment of T2D. It is based on a particular technology that involves the co-formulation of the active ingredient semaglutide with a gastric absorption enhancer called salcaprozate sodium (SNAC); SNAC has a protective action towards digestive enzymes and this facilitates its absorption in the stomach allowing the drug to express its effectiveness.

These molecules have radically changed the T2D management, as beyond increasing insulin secretion, they retain many other beneficial effects for T2D, including reductions in appetite which may favor body weight loss, lowering glucagon secretion and delaying gastric emptying, reduce inflammation, and, not least, improve cardiovascular health (Table 3). Moreover, GLP-1 displayed the capacity to reduce blood pressure and improve dyslipidemia, particularly by reducing triglycerides levels in T2D patients [10,11,12].

Instead, data on the effect of GLP-1R agonists regarding patients with reduced left ventricular function or heart failure are still controversial, as semaglutide has been found to reduce HF and the risk of cardiovascular events in non-diabetic patients with obesity, whereas liraglutide and exenatide seem to increase hospitalization in diabetic patients with HF and a reduced ejection fraction [13,14,15].

Overall, GLP-1RAs substantially lower *glycated haemoglobin* (HbA1c) by up to 1.9% and weight by 2.5–5 kg with favorable effects on cardiovascular disease (CVD), myocardial infarction (MI) and cerebrovascular accident prevention with a low risk of hypoglycemia, which give a great advantage over traditional drugs [16].

Whether severe hypoglycemia is rare (except when used in combination with a sulfonylurea), gastrointestinal (e.g., nausea, vomiting and diarrhea) may occur as adverse events, generally mild-moderate and reduced after the first few months of therapy [17]. Moreover, although in vitro findings suggest cancer inhibition (by modulation cell growth, apoptosis, and angiogenesis) of GLP-1, doubts about potential tumorigenesis are still to be clarified, as liraglutide has been found to be associated with increased incidence of some types of cancer (e.g., breast, thyroid, and pancreatic carcinomas) [18]. However, there is still a lack of knowledge about the increased cancer risk related to GLP-1RA administration from meta-analyses studies.

## 3. Cardiovascular Effects of GLP-1 Receptor Agonists: Preclinical Studies

Native GLP-1 as well as GLP-1RAs may exert different direct and indirect effects leading to reduced inflammation, and modulating cardiometabolic pathophysiology. GLP-1RAs can significantly reduce inflammation and oxidative stress in human endothelial cells; in human umbilical vein endothelial cells (HUVECs) the effect of the GLP-1 analog liraglutide upon TNF-α-induced injury was evaluated [19]. In particular, the potential free radical production by liraglutide was evaluated by incubating HUVECs in the presence of CM-H2DCFDA (a non-fluorescent molecule that passively diffuses into cells). Results indicated that oxidative stress induced by TNF-α was markedly inhibited by liraglutide in a dose-dependent manner in HUVECs. Also, the NF-κB activation induced by TNF-α was significantly decreased after liraglutide (30 nM and 300 nM). Moreover, liraglutide significantly reduced the expression of gp91phox and p22phox, that are components of NADPH oxidase; in addition, the mRNA and protein levels of SOD-2 (Mn-SOD) and catalase were significantly upregulated by liraglutide [19].

In mice fed with a 45% high-fat diet (HFD) or a regular chow diet, the cardiac effects of liraglutide were examined: HFD mice developed 46 ± 2% and 60 ± 2% greater body weight relative to regular chow-diet-fed mice with a consequent impaired glucose tolerance, insulin resistance, and cardiac ceramide accumulation by 16 weeks [20]. After one week from the beginning of the liraglutide treatment, there was a reduction in fasting blood glucose observed in lean and obese groups and the treatment also reduced glucose intolerance in HFD mice. Liraglutide improved all insulin-activated signals (IRS1, Akt, GSK3β, and ERK1/2) evaluated in the heart and liver of mice on HFD in comparison with placebo-treated HFD-fed controls. Moreover, after 16 and 32 weeks, HDF mice had reduced metabolic sensing/modulator protein phosphorylated AMPK in the hearts, which returned to normal after 1 week with liraglutide administration. These data highlight how GLP-1RAs may promote activation of cardioprotective pathways improving cardiac function, and relieving insulin resistance, inflammation and monocyte vascular adhesion [20].

In another study the effect of exendin-4, or exendin-4 and exendin 9–39 for 4 weeks on the regulation of calcium handling to understand the mechanisms in a heart failure (HF) rat model MI was evaluated [20]. Four weeks after MI, the rat hearts showed an increase in chamber end-systolic and end-diastolic diameters, a reduced ejection fraction and fraction shortening and exendin-4 partly reversed progressive remodeling, whereas co-administration of exendin-4 with exendin 9–39 mitigated these alterations. Exendin-4 treatment, post-MI, decreased the infarction size, and interstitial fibrosis. After MI, cardiac levels of total eNOS and phosphorylated eNOS were decreased, whereas exendin-4 treatment increased the eNOS and p-eNOS expression and inhibited the Ca^2+^/calmodulin-dependent kinase II pathways [21]. Other recent data evidenced the importance of atrial and ventricular endocardial GLP-1R as key sites of GLP-1 (liraglutide) cardioprotective action in the ischemic mouse heart [22].

The exposure to psychosocial stress can promote the progression of atherosclerosis-based coronary artery disease and cardiovascular adverse events [23]. In apolipoprotein E-deficient (ApoE−/−) mice fed with a high-fat (HF) diet, the beneficial effects and mechanism of exenatide on stress-related vascular senescence and atherosclerosis were investigated by using non-stressed as well as immobilized-stress in mice. The results evidenced that chronic stress increases the growth of atherosclerotic plaque and enhances vascular endothelial senescence. In mice under chronic stress and treated with exenatide, when compared to mice subjected to stress alone, there was a decrease in macrophage accumulation, plaque microvessel density, improvement on plaque collagen volume, and lowered levels of several chemokines. Exenatide also stimulated adipose adiponectin production in preadipocytes and decreased oxidative stress [24].

Bose et al., using both isolated perfused rat heart and whole animal models of ischemia/reperfusion, have hypothesized that native GLP-1 could directly protect the heart against such injury. In fact, the results revealed that GLP-1 administered before ischemia led to a significant reduction in infarction compared with an inhibitor of its breakdown or saline groups, whereas GLP-1 receptor antagonist exendin (9–39) appears to inhibit the action of GLP-1; several multiple pro-survival kinases are essential for cardioprotection mediated by GLP-1 (cAMP inhibitor Rp-cAMP and PI3K inhibitor LY294002 and by the p44/42 mitogen-activated protein kinase inhibitor UO126) [25].

**Figure 1:**
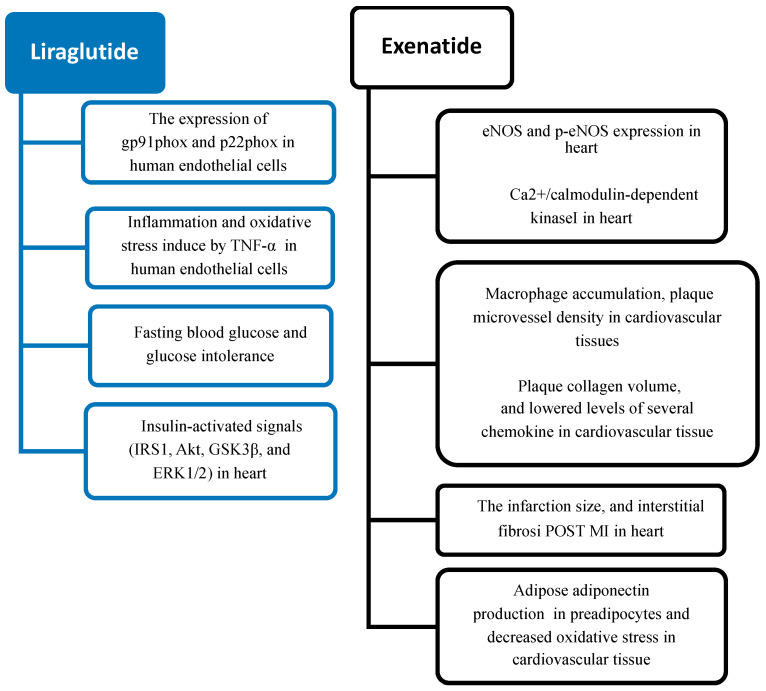
Summary of the GLP-1RA in preclinical studies.

In conclusion, increasing data from preclinical studies strongly suggest how GLP-1R agonists may exert beyond glucose regulation a number of physiological effects, involving multiple organs, that may influence the cardiometabolic risk (Figure 1).

## 4. Cardiovascular Effects of GLP-1 Receptor Agonists: Clinical Studies

Several cardiovascular clinical studies (CVOTs) on GLP-1 receptor agonists have shown that these agents, as well as being safe, are able to significantly reduce the risk of individual major adverse cardiovascular events (MACE), all-cause mortality, hospitalization for heart failure, as well as to slow worsening of renal function in patients with T2D. CVOTs were primarily designed to establish cardiovascular safety by demonstrating non-inferiority to placebo in addition to standard of care, without significant differences in blood glucose. Interestingly, not all the GLP-1RA have demonstrated cardiovascular benefits which would make us think that the cardioprotection of GLP-1RA is not always a class effect but an effect of the individual molecule.

All these CVOTs were of considerable size (>3000 patients) and were performed on cardiovascular-stable patients except for the ELIXA study [26]. In fact, this last study randomized T2D patients who had had a MI or who had been hospitalized for unstable angina within the previous 180 days to receive once-daily lixisenatide (20 µg) or placebo, in addition to locally determined standards of care. The study aimed to demonstrate the non-inferiority or the superiority of lixisenatide to placebo for the primary composite endpoint of cardiovascular death, MI, stroke, or hospitalization for unstable angina. After 25 months in approximately 7000 patients who underwent randomization, the results showed the noninferiority of lixisenatide to placebo (*p* < 0.001) but did not show superiority (*p* = 0.81) for the primary end-point; moreover, there were no significant between-group differences in the rate of hospitalization for heart failure (hazard ratio in the lixisenatide group, 0.96; 95% CI, 0.75 to 1.23) or the rate of death (hazard ratio, 0.94; 95% CI, 0.78 to 1.13). Importantly, lixisenatide achieved these results without causing serious adverse events such as severe hypoglycemia, pancreatitis, pancreatic neoplasms, or allergic reactions than placebo.

The EXSCEL study randomized the highest number of T2D patients (14,752) with (10,782 [73.1%]) or without previous cardiovascular disease, to receive subcutaneous injections of extended release exenatide at a dose of 2 mg or matching placebo once weekly [27]. Also, in this study, the primary composite outcome was the first occurrence of death from cardiovascular causes, nonfatal MI, or nonfatal stroke and the co-primary hypotheses were that exenatide, administered once weekly, would be non-inferior to placebo for safety and superior to placebo with respect to efficacy. After a follow-up of approximately 4 years, the results showed that the incidence of major adverse cardiovascular events did not differ significantly between patients who received exenatide and those who received placebo in patients with or without previous cardiovascular events.

The LEADER study focused on liraglutide (administered subcutaneously once daily) compared to placebo in a cohort of patients with previous cardiovascular events [28]. The primary hypothesis was that liraglutide would be non-inferior to placebo regarding the primary outcome (the first occurrence of death from cardiovascular causes, nonfatal myocardial infarction, or nonfatal stroke). The results were surprising because they showed that the rates of nonfatal MI, nonfatal stroke, and hospitalization for heart failure were significantly lower in the liraglutide group than in the placebo group and the main driver for the cardiovascular benefit of liraglutide was the reduction in cardiovascular mortality (−22% of the relative risk).

SUSTAIN-6 and PIONEER-6 are the two trials that used GLP1RA once-weekly semaglutide (0.5 mg or 1.0 mg) and once-daily semaglutide (3 mg, 7 mg, 14 mg), respectively [29,30]. In both studies, where the majority of patients included had established CVD, the primary composite outcome was the first occurrence of cardiovascular death, nonfatal myocardial infarction, or nonfatal stroke and the hypothesis that semaglutide would be noninferior to placebo for the primary endpoint. These studies concluded that the cardiovascular risk profile of once-weekly subcutaneous and once-daily oral semaglutide was not inferior to placebo, while even in weekly semaglutide the rate of cardiovascular death, nonfatal myocardial infarction, or nonfatal stroke was significantly lower among patients receiving semaglutide than among those receiving placebo. The main driver for the cardiovascular benefit of weekly semaglutide was the reduction in non-fatal stroke (−39% of the relative risk in the SUSTAIN6 study).

In the HARMONY OUTCOMES the safety and efficacy of once-weekly albiglutide (30–50 mg, based on glycemic response and tolerability) was evaluated according to the prevention of cardiovascular mortality, myocardial infarction, or stroke in patients with T2D and cardiovascular disease [31]. It was hypothesized that albiglutide was non-inferior to placebo for these outcomes, but the results indicated that albiglutide was superior to placebo for major adverse cardiovascular events (MACE) with a 25% reduction in the relative risk for fatal or non-fatal MI.

In the AMPLITUDE-O trial, efpeglenatide added once weekly at a dose of 4 or 6 mg to current therapy in patients with T2D and either a history of CVD or current kidney disease plus at least one other cardiovascular risk factor reduces cardiovascular risk compared to placebo, showing also, in this case, the superiority of GLP1RA therapy [32].

The REWIND study was conducted in patients with T2D who had a previous cardiovascular event or cardiovascular risk factors and were randomly assigned (1:1) to a weekly subcutaneous injection of dulaglutide (1.5 mg) or placebo [33]. The primary outcome was the first occurrence of the composite endpoint of non-fatal myocardial infarction, non-fatal stroke, or death from cardiovascular causes (including unknown causes), which was assessed in the intention-to-treat population. It should be noted that REWIND is the first cardiovascular outcome study designed to document the superiority, and not the non-inferiority, of GLP-1RA treatment compared to standard care. After a follow-up of a mean of 5.4 years, treatment with dulaglutide was associated with a lower incidence of adverse cardiovascular events than placebo in patients with T2D with and without prior CVD. These results confirm the protective cardiovascular effect of dulaglutide in people with T2D, extending its benefits to a wider and more heterogeneous population. Only 31.5% of participants had a known history of CVD at the time of recruitment, while the majority had only cardiovascular risk factors and were therefore in primary prevention. The REWIND population also differs from that of previous cardiovascular outcome studies with GLP-1RA due to the large presence of a female population (46% vs. 30–40% in the other studies), a lower median HbA1c value (7.2% vs. 7.7–8.7% in the other studies) and a longer follow-up (5.4 years vs. 1.6–3.8 years in the other studies).

## 5. SGLT2 Inhibitors

It has been thought for a long time that the liver was the exclusive site of glucose production in the post-absorptive state in humans, whereas now it is known that also the kidney is able to reabsorb glucose from the glomerular filtrate through the sodium-glucose co-transporters (SGLT) 1 and 2 placed under the Bowman’s capsule at the proximal convoluted tubule. This mechanism preserves essential energy substrates for the organism. The maximal renal glucose reabsorption capacity (TmG) is higher in diabetics than normal subjects and contributes to the hyperglycemic state in the absence of glycosuria. Over the years, therefore, numerous attempts have been performed by using drugs which, targeting the kidney, could increasingly promote glycosuria and lead to euglycemia (Table 4) [34].

Sodium-glucose transporter inhibitors (SGLT2i) act by inhibiting the sodium-glucose co-transporter SGLT2, expressed almost exclusively in proximal tubules, which mediates the block of the reabsorption of glucose and sodium and allow their elimination through the urine with a non-insulin-dependent mechanism, leading to a reduction in blood glucose levels, weight, and blood pressure without the risk of hypoglycemia. Recently clinical studies conducted with SGLT2 inhibitors in subjects with T2D have demonstrated the ability of this class of drugs to achieve benefits at the cardiac and renal level due to their capacity to reduce the risk of hospitalizations for heart failure and the progression of the renal damage. The favorable mechanisms of these agents are likely mediated almost in part by their natriuretic effect, which is associated with the glycosuric effect that, with the glomerular hyperfiltration, reduces the activation of the renin-angiotensin-aldosterone system.

Thus, a lot of evidence is emerging on the beneficial effects of SGLT2i on cardiovascular [35,36] and renal [37] risk and outcomes in people with T2D. Nonetheless, the benefits on glycemic control, blood pressure, body weight, lipid profiles and renal function could not justify the excess of the predicted benefits associated with improvements in cardiovascular risk factors when a patient is treated with SGLT2i. As a result, several authors have suggested other mechanisms by which these drugs may indirectly and directly improve cardiovascular and renal outcomes.

Regarding the effect of SGLT2i on decrease in body weight, it was initially believed that it was due almost exclusively to the diuretic effect of these drugs. A study with dapagliflozin used as an add-on to metformin, however, has documented, through the use of the dual x-ray absorptiometry (DEXA), that these was an effective reduction in fat mass, quantified as a real loss of adipose visceral and subcutaneous tissue [38].

SGLT2i, through the mechanism of diuresis and natriuresis, determines the reduction in plasma volume and therefore lead to the reduction in cardiac preload [39]. This effect of SGLT2i occurs without the increase in sympathetic activity which can be harmful to the heart. Another indirect mechanism of SGLT2-inhibitor-mediated cardiovascular benefits include improved myocardial energy efficiency by shifting to ketone metabolism, a more energy efficient substrate than glucose or fatty acids. Moreover, they may improve cardiac function through action on glycosuria, as the effect of these drugs induces a state of ‘fasting mimicry’ which activates the enzymes with important anti-inflammatory effects within the heart, sirtuin 1 (SIRT1) and adenosine monophosphate-activated protein kinase (AMPK) [40]. SGLT2 inhibition results in an increased delivery of sodium to the macula densa, which leads to renal afferent arteriolar vasoconstriction and reduces the hyperfiltration that characterizes the early stages of diabetic kidney disease stabilizing kidney function over time [40]. However, it is not certain whether the improvement in endothelial function is a direct effect of these drugs, or their indirect effect linked to the improvement of metabolic control.

Moreover, a direct cardiovascular beneficial effect of SGLT2i treatment includes the myocardial Na/H pump inhibition which improves sensitivity to endogenous natriuretic peptides and reduces cardiac hypertrophy, fibrosis and remodeling [41].

SGLT2 inhibitors also have an important effect on lipid profile at different levels; they reduce lipid accumulation and visceral and subcutaneous fat, modulate key molecules in lipid synthesis and transportation and the oxidation of fatty acids, and shift substrate utilization from carbohydrates to lipids and ketone bodies [42].

Interestingly, recent data shows possible use of dapagliflozin for transthyretin amyloid cardiomyopathy, in terms of tolerability and changes in NT-proBNP levels [43].

The SGLT2i currently available are dapagliflozin 10 mg, canaglifozin 100 mg and 300 mg, empagliflozin 10 mg and 25 mg and ertugliflozin 5 mg and 25 mg (Table 4). They can be administered orally as monotherapy or in combination with other drugs such as metformin or pioglitazone or DPP4 inhibitors (Table 5). There are several studies on the effectiveness of SGLT2 inhibitors on glycemia and weight loss in monotherapy or as additional therapy to other oral hypoglycemics or insulin therapy [44,45]. The average reduction in HbA1c was found to be between 0.6–1.2% with maintenance of this glycemic control for a long time (until the 104th week) [46].

Regarding the effect of SGLT2 inhibitors on weight loss, a meta-analysis, including most of the available studies on these drugs, in comparison with other hypoglycemic drugs or placebo, has documented a significant weight loss of 1.74 kg vs. placebo and 1.11 kg vs. active treatments [47]. This phenomenon is probably related to the glycosuric effect of these drugs which leads to osmotic diuresis or causes a caloric loss calculated at around 200–300 kcal per day.

## 6. Cardiovascular Effects of SGLT2 Inhibitors: Preclinical Studies

In diabetic (db/db) and wild type (WT) mice, cardiomyopathy was induced by subcutaneous infusion of angiotensin II by using an osmotic pump and concomitant SGLT2I (dapagliflozin) administration in drinking water; whether angiotensin II infusion developed cardiac hypertrophy, myocardial fibrosis and inflammation, administration of SGLT2I decreased glucose concentration and attenuated fibrosis and inflammation [48]. Moreover, dapagliflozin improve the left ventricular fractional shortening in db/db mice treated with angiotensin II, showing cardioprotective effects, which may open new pharmacologic strategies for the treatment of diabetic cardiomyopathy [48]. Adingupu et al. investigated whether the treatment with SGLT2i (empagliflozin) could provide benefits in terms of metabolic and cardiac dysfunction in ob/ob−/− mice, where the leptin deficiency promotes metabolic syndrome, diabetes, and hepatic steatosis, as well as cardiac dysfunction [49]. Results evidenced that SGLT2i administration may improve glucose and lipid metabolism whereas glucagon/insulin ratio and ketone levels were increased. At the liver level, the treatment reduced alanine aminotransferase, liver triglyceride and steatosis. Regarding cardiac and endothelial function, SGLT2i improved coronary microvascular function and cardiac contractile function and L-Arginine/ADMA ratio, a marker of endothelial function, increased [49]. In Zucker fatty diabetic (ZDF) rats, the effects of empagliflozin on β-cell function, glucose toxicity, endothelial dysfunction, oxidative stress, AGE/RAGE signaling and inflammation was investigated. Before SGLT2i treatment, blood glucose was higher in all ZDF rats compared to the lean control rats; after SGLT2i treatment (6 weeks), fasting blood glucose levels were decreased by about 40% and endothelial function (thoracic aorta), glucotoxicity, inflammation and reduced oxidative stress status improved [50]. Always in Zucker diabetic fatty rats, the effect of SGLT2 inhibitors on the whole cardiac metabolic profile was evaluated by using the untargeted metabolomics technique; treatment for six weeks with empagliflozin reduced the cardiac content of sphingolipids (ceramides and sphingomyelins) and glycerophospholipids and decreased the cardiac content of the fatty acid transporter cluster of differentiation 36 (CD36) [51]. Also, metabolites such as glutamic acid, gamma-aminobutyric acid and sarcosine, implicated in the metabolic control of the cardiac function, were modulated by empagliflozin, which demonstrated protective effects in terms of cardiometabolic diseases whose development is related with consistent cardiac metabolome and lipidome dysregulation [51]. Uthman et al., have studied the ability of SGLT2i empagliflozin (EMPA) and dapagliflozin (DAPA) to reduce tumor necrosis factor α (TNFα)-induced endothelial inflammation in vitro [52]. In particular, reactive oxygen species (ROS) and nitric oxide (NO) were measured using live cell imaging after incubation with EMPA or DAPA in TNFα-stimulated human coronary arterial endothelial cells (HCAECs) and human umbilical vein endothelial cells (HUVECs). EMPA and DAPA improved intracellular NO and ROS levels in TNFα-stimulated HCAECs. In both cell types, SGLT2i administration does not affect the increase in adhesion molecule expression (VCAM-1 and ICAM-1), which was elevated after 4 h TNFα exposition [52]. Thus, the restoring of NO bioavailability seems to derive more from the inhibition of ROS generation than by affecting eNOS expression or signaling, barrier function and adhesion molecules expression.

Liu et al. study focused on the role of SGLT2i (empagliflozin) regarding the progression of atherosclerosis in a non-diabetic model mice [50]. In particular, mice were fed with a Western diet for 12 weeks to induce atherosclerosis. Then, during the 7 h week, a group of mice was treated with drinking water containing empagliflozin (EMPA group) and another group was treated with normal water and Western diet (AS). After 12 weeks, the entire aorta was harvested for each group for the determination of atherosclerotic lesion size and area and evaluation of the serum lipid profile. Empagliflozin decreased the levels of triglyceride, total cholesterol and LDL in the EMPA group vs. AS group. However, HDL was not significantly different between groups. Interestingly, the atherosclerotic lesion area in the aortic arch was decreased in the EMPA group compared with the AS group [53].

Recent data suggest that dapagliflozin may exert cardioprotection against myocardial ischemia/reperfusion injury beyond its hypoglycemic effect; in fact, high-dose dapagliflozin pretreatment (40 mg/kg/day started 7 days before the cardiac I/R intervention) decreased infarct size and biomarkers of cardiac damage (cTnI, CK-MB, and LDH) and relieved NLRP3 inflammasome activation (reduced IL-1β blood levels, reduced expression of myocardial inflammation-related proteins, and inhibition of cardiac caspase-1 activity) in male C57BL/6 mice. Moreover, dapagliflozin (10 μM) directly acts on cardiomyocytes by increasing NHE1/NCX expression [54].

**Figure 2:**
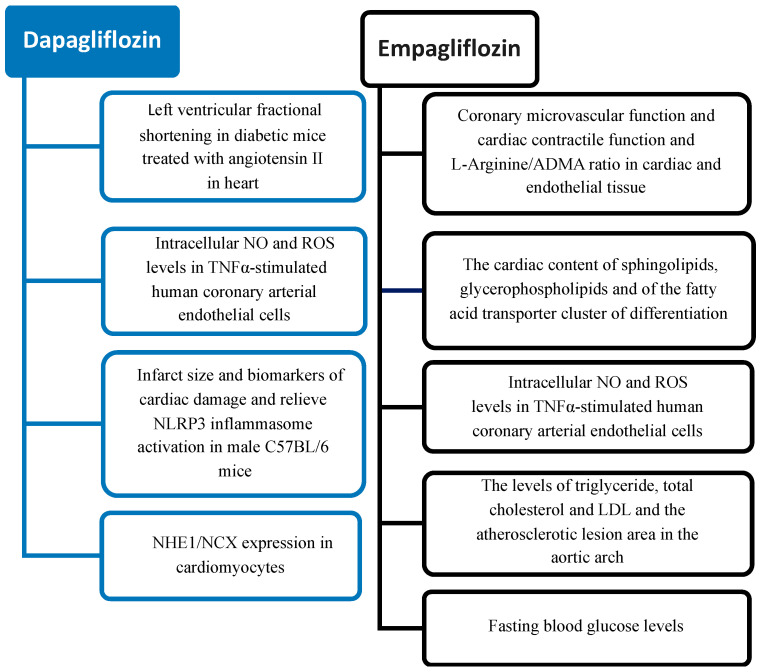
Summary of the SGLT2i in preclinical studies.

A summary of the SGLT2i effects suggested by results obtained in preclinical studies is reported in Figure 2.

## 7. Cardiovascular Effects of SGLT2 Inhibitors: Clinical Studies

In recent years, several studies have been conducted on cardiovascular safety or benefit using SGLT2i molecules (empagliflozin, dapagliflozin, canagliflozin, ertugliflozin). These studies differ in the number of participants (between 3730 and 17,160), study duration (between 16 months and 4.2 years), presence of heart failure or renal disease, if baseline heart failure is characterized by reduced ejection fraction (HFrEF) or preserved ejection fraction (HFpEF), associated comorbidities or risk factors, which clearly confer a variable risk and rate of cardiovascular or renal events during the study.

The EMPAREG OUTCOME study was the first study using an anti-hyperglycemic drug (empagliflozin) to demonstrate a benefit on cardiovascular mortality in patients with T2D [55]. This is a multicenter randomized double-blind study where a placebo compared with an empagliflozin arm at a dosage of 10 mg or 25 mg, involved 7020 patients with T2D at high cardiovascular risk (previous heart attack, coronary artery disease, heart failure, stroke), with acceptable glycemic compensation (HbA1c at baseline of approximately 8%), with diabetes diagnosed for more than ten years and for an average observation duration of over three years. The patients were already on standard hypoglycemic treatment and were on optimal treatment with ace-inhibitors or sartans, statins and aspirin (ASA, non-steroidal anti-inflammatory drug). The primary endpoint was three-point major adverse cardiovascular events (MACE, cardiovascular mortality, non-fatal heart attack, non-fatal stroke). The secondary outcome was the composite of the primary outcome plus hospitalization for unstable angina. The results were surprising for both the empagliflozin dosages: patients treated with empagliflozin in association with standard therapy showed a significant (14%) reduction in cardiovascular risk, significant (38%) reduction in cardiovascular mortality, significant (32%) reduction in all-cause mortality, and significant (35%) reduction in hospitalization for heart failure. In addition, empagliflozin showed an efficacy and safety profile with the reduction in glycated hemoglobin without increasing the risk of hypoglycemia, weight and blood pressure reduction and a small increase in LDL and HDL cholesterol. The increase in genital infections caused by the drug was overall well tolerated.

The efficacy of empagliflozin in chronic heart failure occurred through two main studies [56,57]. The first to be published was the EMPEROR-Reduced study which explored the efficacy of empagliflozin in the treatment of heart failure with reduced ejection fraction (HFrEF) in HFrEF patients hospitalized in NYHA functional class II-IV, clinically stable for at least 4 weeks, with glomerular filtration rate (GFR) >20 mL/min/1.73 m^2^ and elevated N-terminal fragment values of B-type natriuretic propeptide (NT-proBNP). The patients were randomized double-blind to receive empagliflozin 10 mg/day or placebo, in addition to standard treatment of HFrEF. The primary objective of this study was a combined endpoint of cardiovascular mortality and hospitalization for heart failure and as secondary objectives the total number of hospitalizations for heart failure (including first event and recurrences) and the change in estimated GFR (eGFR).

The DECLARE (Dapagliflozin Effect on Cardiovascular Events)-TIMI 58 appears to be the largest metabolic outcome study (CVOT) evaluating SGLT2i effects to date [58]. This study compared the results of dapagliflozin on cardiovascular events versus placebo. It is a multi-center and multi-ethnic study, conducted over a five-year period and involving more than 17,000 adults suffering from type 2 diabetes (T2D) who had multiple risk factors for cardiovascular disease or who had already been diagnosed with CVD.

In the DECLARE-TIMI 58 study, dapagliflozin achieved its primary safety endpoint of non-inferiority for major adverse cardiovascular events (MACE), a statistically significant reduction in the composite endpoint of hospitalization for heart failure (hHF) or cardiovascular death (hazard ratio [HR] 0.83, 95% CI 0.73–0.95, driven by a reduction in hHF) and a lower incidence of MACE events but without reaching statistical significance. The renal-specific outcome was significantly reduced with dapagliflozin (HR 0.53, 95% CI 0.43–0.66). The results of this study are particularly relevant because the subjects examined essentially represent almost all patients with T2D and not just those with a history of cardiovascular disease, a preferential object in studies already conducted with other SGLT-2 inhibitors. These benefits on a larger scale suggest the opportunity to use dapagliflozin already in the initial phase of the treatment of T2D as primary prevention of CVD, and not only after a cardiovascular event has already occurred (as secondary prevention).

DAPA-HF [59] is the first clinical trial conducted with an SGLT2 inhibitor in a population of HfrEF patients, with and without T2D. Study results showed that dapagliflozin, added to standard of care compared to placebo in a population of patients with stable heart failure (from NYHA II at IV) and reduced ejection fraction (LVEF less than or equal to 40%), significantly reduced the risk of the primary composite endpoint of cardiovascular death or worsening of heart failure (defined as hospitalization or an urgent visit) by 26% (*p* < 0.0001). The results also demonstrated a reduction in each of the individual components of the composite endpoint.

In this randomized, placebo-controlled trial involving patients with heart failure and a reduced left ventricular ejection fraction, the risk of the primary composite outcome of worsening heart failure (hospitalization or an urgent visit resulting in intravenous therapy for heart failure) or death from cardiovascular causes was lower in the dapagliflozin group than in the placebo group. Each of the three components of the composite outcome was less common in the dapagliflozin group, as were the total numbers of hospitalizations for heart failure and deaths from cardiovascular causes. Dapagliflozin was as effective in 55% of patients without T2D as in those with. This demonstration of the cardiovascular benefits of an SGLT2 inhibitor in patients without diabetes provides support for prior suggestions that such treatment has beneficial actions other than glucose lowering and extends the therapeutic role of dapagliflozin beyond patients with diabetes.

The Dapagliflozin Evaluation to Improve the LIVEs of Patients With Preserved Ejection Fraction Heart Failure (DELIVER) trial is testing the hypothesis that the SGLT2i dapagliflozin (10 mg once daily vs. placebo) can reduce cardiovascular death and heart failure hospitalization in patients with heart failure with a LVEF > 40% (HFpEF and heart failure with midrange ejection fraction-HFmrEF), with or without diabetes [60]. In this study, dapagliflozin reduced the composite outcome of cardiovascular death or worsening of heart failure by 18% (*p* < 0.001, 16.4% in the dapagliflozin group and 19.5% in the placebo group). All individual components contributed to the superiority of the primary endpoint. The results were consistent in the main subgroups examined and extended the benefits of dapagliflozin to the entire spectrum of patients with heart failure regardless of the value of LVEF. Furthermore, the safety of using dapagliflozin was also confirmed in this patient population as there were no differences between dapagliflozin and placebo regarding adverse events.

The CANVAS study [61] collects data relating to the integrated analysis of the CANVAS and CANVAS-R trials. This program evaluated the efficacy, safety and durability of canagliflozin in over 10,000 patients with T2D with a previous history of cardiovascular disease or who had at least two of the cardiovascular risk factors. Canagliflozin achieved a 14% reduction in the risk of the composite primary endpoint of cardiovascular mortality, non-fatal MI, or non-fatal stroke (HR: 0.86; 95% CI: 0.75 to 0.97). It also demonstrated its superiority and safety in the case of cardiovascular disease (*p* < 0.0001 for non-inferiority) compared to a placebo (*p* = 0.0158). Further analyses also revealed that canagliflozin in patients with T2D also reduced the risk of hospitalization for heart failure by 33% (HR: 0.67; 95% CI: from 0.52 to 0.87). Additionally, diabetic patients treated with canagliflozin had potential renal protective effects, delaying the progression of albuminuria and reducing the risk of clinically important renal outcomes (e.g., renal death, renal replacement therapy, and a 40% reduction in eGFR) by 40% (HR: 0.60; 95% CI: 0.47 to 0.77).

The VERTIS CV trial [61] was a randomized, double-blind, placebo-controlled, cardiovascular outcomes trial that evaluated the cardiovascular efficacy and safety of ertugliflozin in adults with T2D and established atherosclerotic CVD. The 8000 patients enrolled were randomized in a 1:1:1 fashion to either ertugliflozin 5 mg, 15 mg, or matching placebo. The primary outcome (composite of cardiovascular death, non-fatal MI and non-fatal stroke), occurred in 11.9% of patients in the ertugliflozin groups and 11.9% of patients in the placebo group (HR 0.97, 95% CI 0.85–1.11; *p* < 0.001 for non-inferiority), demonstrating non-inferiority of ertugliflozin, whereas superiority would be tested for “key” secondary outcomes (composite of cardiovascular death and hospitalization for heart failure, mortality from cardiovascular causes and a “renal” composite such death from renal causes, need for replacement therapy, doubling of blood levels creatinine). The composite outcome of cardiovascular death and hospitalization for heart failure occurred in 8.1% of subjects in the ertugliflozin groups and in 9.1% of patients in the placebo group (HR 0.88, 95% CI 0.75–1.03; *p* = 0.11 for superiority). The HR (ertugliflozin vs. placebo) was 0.92 (95% CI 0.77–1.11) for cardiovascular mortality and 0.81 (95% CI 0.63–1.04) for the composite outcome renal, in both cases not significant for superiority. Regarding adverse events, there was a greater number of genitourinary infections, distal amputations, and cases of diabetic ketoacidosis in the ertugliflozin groups compared to the placebo group, without any difference regarding the incidence of hypovolemia, fractures and severe hypoglycemia.

## 8. Innovative Therapeutic Developments in the T2D Scenario

Despite detailed and refined protocols for diabetes treatment developed in the last years, many of the underlying mechanisms remain to be further clarified, still limiting early intervention and prevention and treatment of complications. New technologies may represent promising tools for enhancing knowledge of important aspects for the disease treatment and prevention of cardiovascular complications, increasing wellness and life quality of patients, which can be addressed as innovative therapeutic frontiers.

We discuss some recent new exciting therapeutic options related to technological and knowledge developments in the field, such as cellular senescence in beta cells, epigenetic modifications and non-coding RNA, gut microbiome profile, mitochondrial dynamics. These tools are so transformative to have the potential to change the future of T2D management, improving the tailoring care for the patient according to the specific personal needs of each patient.

### 8.1. β-Cells Senescence in Type 2 Diabetes

Cellular senescence is a process characterized by a cell cycle arrest and cell alterations (e.g., oxidative stress, telomere shortening, mitochondrial dysfunction, DNA damage), although the cells remain metabolically active, thus releasing proinflammatory cytokines, chemokines, growth regulators, angiogenic factors, and matrix metalloproteinase that represent the senescence-associated secretory phenotype (SASP) [62].

It is known that metformin improves insulin sensitivity and reduces glucose levels and inhibits liver gluconeogenesis, acting through inhibition of the mitochondrial respiratory-chain complex I and glycerophosphate dehydrogenase as well as by activating adenosine monophosphate-activated protein kinase (AMPK) [63,64]. Moreover, some data suggested that metformin is also able to affect pathophysiological pathways associated with chronic diseases (inflammation, oxidative stress, protein glycation, cell senescence, apoptosis) [65].

In particular, among putative cellular mechanisms related to its action in senescence, metformin reduced p16 and p21 protein levels and the inflammatory cytokines and oncogenes that belong to SASP; one key additive mechanism by which metformin may modulate senescence has been identified in the upregulation of DICER1, a key enzyme that processes microRNAs [66,67]. Other data indicated that metformin improves the progression of atherosclerosis (plaque area) and vascular senescence (senescence-associated β-galactosidase activity) and prevents the upregulation of angiotensin II type 1 receptor induced by the high-fat diet in the aortas of mice [68]. Always in relation to senescence, metformin has been proven to enhance B-cell function and antibody responses, contributing to improving humoral immunity and reducing inflammation and autoimmunity in elderly T2D patients [65].

Nonetheless, other anti-diabetic drugs showed anti-senescence properties; GLP-1 reduces ROS production, likely through mechanisms involving downstream protein kinase A (PKA) signaling and upregulation of antioxidant genes in both endothelial cells and in diabetic rat models [69]. GLP-1 also reduced the oxidative stress levels through multiple different mechanisms: stimulation of GLP-1 receptor increased levels of cAMP in the cell, reducing levels of ROS produced by nicotinamide adenine dinucleotide phosphate-oxidase (NOX, a membrane-bound superoxide-producing enzyme) and xanthine oxidase (XO, a superoxide-producing enzyme) [66]. The decrease in NOX activity is also induced by GLP-1 inactivation of the protein kinase C (PKC). Increased cAMP level by GLP-1R activation can increase PKA activity (decreasing the Rho/ROCK pathway and reducing ROS levels), and lower the activity of Src kinase, which is an activator of NOX (lowering superoxide production). Moreover, reduction in advanced glycation end-product/expression of advanced glycation end-product receptors (AGE/RAGE) can lower XO, NOX and mitochondrial ROS production. Increased antioxidant capacity favors redox homeostasis and has been shown to be induced by GLP-1R activation and/or increased Nrf2 concentration or expression by GLP-1. Lowering ROS can affect many cellular pathways (e.g., PI3K/Akt, p38 MAPK and JNK) and decrease inflammatory (reduced NF-κβ activity) and apoptotic pathways [70].

Other drugs used in the cardiovascular setting have been proven to oppose senescence: ASA increases nitric oxide bioavailability and reduces oxidative stress, upregulating telomerase activity and delaying senescence in endothelial cells; statins (cholesterol level lowering drugs) preserve telomere length, upregulate endothelial nitric oxide synthase, SIRT1, and inhibit the mTOR signaling pathway; angiotensin-converting enzyme (ACE) inhibitors and angiotensin II receptor blockers (ARBs, losartan) prevent DNA damage and preserve telomere integrity affecting the oxidative stress status [71,72,73,74,75,76]. Rapamycin is an inhibitor of mTOR pathway, with important effects on several key cellular pathways (e.g., cell growth, synthesis of proteins, ribosomal biogenesis, transcriptional regulation, glucose and lipid metabolism, mitochondrial dysfunction, cell senescence and the hypertrophic phenotype) [77].

Another typicity of senescent cells is the resistance to apoptosis; pharmacological attempts that target these anti-apoptotic pathways have led to the development of senolytic drugs (including drugs, plant extracts, or peptides such as dasatinib, quercetin or FOXO4-DRI, respectively, designed to selectively remove senescent cells) [78,79]. In particular, the combination of dasatinib plus quercetin (D + Q) was found effective to exert a senolytic effect by inhibiting several kinases (D: tyrosine kinase inhibitor targeting senescent cellular anti-apoptotic pathways, including p53 and p21 pathways; Q: a flavonoid targeting several senescent cellular anti-apoptotic pathways, as the PI3K, AKT, and HIF-1α pathways) [62].

Foxo4-DRI, a peptide that inhibits binding FOXO4 with p53, induced p53-dependent senolysis and improved phenotypes in fast-aging models (XpdTTD/TTD) and in naturally aged mice [80]. Moreover, administration of ABT263 (Navitoclax), ABT199 (Venetoclax) and ABT737, which target the BCL-2 pathway, eliminates senescent pancreatic β cells in experimental models [81,82,83]. Altogether, these results open new perspectives in the use of these drugs as anti-senescence tools, as well as in terms of innovative therapies for prevention of T2D and its complications.

Although senescence of hepatic cells (e.g., hepatocytes and hepatic stellate cells, mainly driven by mitochondrial dysfunction, altered lipid metabolism, liver fat accumulation), adipose tissue (dysfunctional cells, increased inflammation, decreased insulin sensitivity and lipid storage) and muscle cells (mainly by through increased oxidative cells and mitochondrial dysfunction) may contribute to T2D development, cellular senescence of pancreatic β-cells is emerging as one major determinant for T2D etiopathogenesis [62,84,85].

Different pathways have been related to pancreatic β-cell senescence, as well as with the pathogenesis and development of diabetes. Development and worsening of T2D and β-cell senescence appear related to an increase in SASP biomarkers (e.g., proinflammatory cytokines and chemokines). Moreover, many other markers that may contribute to β-cell senescence are emerging, which may represent efficacious targets of specific pharmacological strategies [62]. Cell cycle regulation (progression from the G to the S phase) is dependent on cyclins binding their respective cyclin-dependent kinases (CDKs), inducing cell proliferation, which in turn are modulated by cyclin/CDK complex inactivation and inhibition through cyclin-dependent kinase inhibitors (CDKIs) [86]. Thus, CDKI overexpression may induce β-cell senescence; conversely, loss of CDKI or their epigenetic regulators led to stimulation of pancreatic beta-cell proliferation. Targeting CDKI (e.g., through inhibition of this system, for example with palbociclib) might represent a therapeutic development to study for enhancing β cell number [87].

Another pathway which affects cell cycle in pancreatic β cells in the one related to multiple endocrine neoplasia type 1 (MEN1) tumor suppressor gene; menin, encoded by the MEN1 gene, is a critical suppressor controlling pancreatic beta cell proliferation and mass, whereas deletion of the Men1 gene increased beta cell proliferation in mice (as Men1 inactivation might alter expression of p18, p27, and other established cell cycle regulators like CDK2 and CDK4 in endocrine cells) [88]. Recent data showed that menin inhibitors (MI-463 and MI-503) epigenetically regulates the menin/JunD/Pbk axis, revealing a new (suppressive) mechanism of action useful to develop future drugs for treatment of diabetes, acting through the modulation of the beta cell mass and improvement of glucose control [89].

The oncoprotein Wip1 is an inhibitor for p38 MAPK; interestingly, wild-type p53-inducible phosphatase 1 (Wip1) knockout induces insulin resistance in mice, whereas Wip1 induction reduces p38 MAPK activation, increasing in turn β-cell proliferation, thus providing a new line of investigation as a possible candidate target also for T2D innovative therapeutic approaches [90,91].

It is interesting that some recent data suggested that subpopulations of senescent tumor cells have the potential to re-enter the cell cycle by escaping or reverting from the senescent state under the influence of specific treatments (e.g., etoposide or doxorubicin) [92]. In this case, senescence is a status by which cancer cells aim to face therapies and later escape and start dividing again, acquiring a belligerent proliferation phenotype; alternatively in other conditions (e.g., T2D), this characteristic might permit renovation and restore of the tissues. Thus, deletion of senescent β-cells or the reversal of senescence in a subpopulation of aged β-cells may discontinue processes of dysfunction and disease. However, further advances must be made to better understand the molecular basis of cellular senescence in different tissue, characterizing the progression of senescence in β-cells as well in other cell types which also contribute to diabetes, identify more critical drivers of senescence and SASP biomarkers as efficacious candidates for targeted therapies, study the heterogeneity in the action of senolytic drugs, and determine more in details the effect elicited targeting senescent cells. In fact, an important point is to avoid the development of side effects or worsening of concomitant diseases, due to undesirable activation or inhibition of other related cellular pathways.

To note, well-known life-style interventions, such as physical activity and a healthy diet (e.g., adhesion to the Mediterranean diet) may have anti-aging effects and cannot be forgotten as available and exploitable tools to counteract cell senescence [93,94]. In fact, different components of the Mediterranean diet (e.g., polyphenols, beta-carotene, vitamins C and E, omega-3 fatty acids) promote DNA health (reducing DNA damage and protecting telomeres) and consequently wellness and longevity [95,96,97]. In particular, resveratrol, one of the most studied components of the mediterranean diet and a polyphenolic compound contained in many plants (e.g., grapes, berries, cocoa, tomatoes, and peanuts), exert its anti-senescence effects through its anti-oxidative and anti-inflammatory properties and the capacity to activates the expression of various antioxidant defensive enzymes (e.g., heme oxygenase 1, catalase, glutathione peroxidase, and superoxide dismutase) as well as regulating different key signaling pathways (including sirtuin 1, nuclear factor-erythroid 2-related factor 2 and nuclear factor κB) [98,99]. On the other hand, exercise resulted in the health of islet cells, in terms of their function, proliferation, and survival rate [100]. Moreover, physical exercise prevents cellular senescence and protects from stress-induced vascular apoptosis by modulating telomere-stabilizing proteins in mice and in humans [101].

### 8.2. Mitochondrial Dynamics in T2D

T2D is characterized by mitochondrial dysfunction (e.g., abnormal mitophagy, fission, fusion, and biosynthesis), elevated oxidative stress and reduced ATP levels, thus therapeutic agents targeted on the recovery of mitochondrial function are very interesting to develop as new additive pharmacological tools to apply in the T2D setting. The normal function of mitochondria is related to the integrity of a dynamic equilibrium that allows for preserving the mitochondrial morphology, function and distribution as well as to respond and adjust the number and function of mitochondria, guaranteed through two opposing events: mitochondrial fission and fusion [102].

Mitochondrial fission refers to a process through which mitochondria divide or segregate into two organelles (essential for quality control), whereas mitochondrial fusion means that two separate mitochondria can fuse together (allowing the transfer of gene products for optimal functioning) [103]. Mitochondrial fusion is modulated by mitofusin-1 (Mfn1), mitofusin-2 (Mfn2), and optic atrophy 1 (Opa1), whereas mitochondrial fission by dynamin related protein 1 (Drp1), mitochondrial fission 1 (Fis1) and mitochondrial fission factor (MFF); alterations in the expression of these markers have been found related to development of T2D and its complications (e.g., diabetic nephropathy, diabetic cardiomyopathy, diabetic retinopathy, diabetic peripheral neuropathy) [104].

Conventional oral antidiabetic drugs, such as SGLT-2 inhibitors and metformin, exert beneficial effects and improve mitochondrial function by regulating mitochondrial dynamics [105]. In a rat model of insulin resistance and obesity, empagliflozin, a sodium glucose co-transporter-2 inhibitor, regulate mitochondrial dynamics, increasing fusion protein MFN2 expression, while decreasing the levels of the fission protein DRP1 in white adipose tissue [106]. Always in T2D rats, empagliflozin has been shown to mitigate diabetes related atrial fibrillation improving mitochondrial function likely by restoring the decrease in phosphorylated AMPK expression and altered protein levels related to mitochondrial biogenesis and dynamics; empagliflozin also recover mitochondrial function in H9c2 cells cultured with high glucose medium [107,108].

Empagliflozin re-establishes AMP/ATP, activating adenosine monophosphate (AMP)-activated protein kinase (AMPK), inducing Drp1^S637^ phosphorylation and decreasing in Drp1^S616^ phosphorylation, inducing inhibition of mitochondrial fission, thus improving cardiac microvascular injury in T2D mice [109]; other results evidenced that empagliflozin could prevent and/or improve renal ischemia-reperfusion injury through anti-inflammatory effects and the activation of the AMPK-OPA1 pathway, enhancing mitochondrial fusion [110]. In a mice model of retinal ischemia and reperfusion injury, empagliflozin acts on mitochondrial dynamics upregulating the expression of Mfn1 and Opa1, reducing microglia-mediated neuroinflammation [111]. In an experimental model of acute MI in T2D rats, empagliflozin normalized the size and number of heart mitochondria and prevent the diabetes-induced excessive reduction in mitochondrial size through inhibition of Fis1 upregulation and consequent ROS production [112]. Other recent results reported that empagliflozin inhibits mitochondrial fission and improved energy metabolic efficiency in heart failure mice by regulating the expression of mitochondrial dynamics-related proteins, suggesting that empagliflozin-related cardiac beneficial effect may be associated, almost in part, to the normalization of mitochondria and the increase in ATP production [113].

Recent data suggested that dapagliflozin improved mitochondrial dynamics (OPA1, DRP1, and MFN2), together with skeletal muscle mitochondrial biogenesis (PGC-1α, NRF1, TFAM, and COX IV), and mitophagy (PGAM5 and PINK1) related protein levels in T2D rats [114].

Moreover, dapagliflozin induced improvement of fusion-fission proteins (Mfn-1, Mfn-2, and Fis-1) in a rat model of metabolic syndrome [115].

Sotagliflozin is able to improve mitochondrial fission and reactive oxygen species production in a metabolic-syndrome-related rat model of heart failure with preserved ejection fraction [116] Ipragliflozin is able to restore the levels of Opa1 and Mfn2 to their normal values in an experimental model (rats under a high-fat diet) [117].

Other findings indicate that metformin may increase mitochondria efficiency and reduce oxidative damage; in particular, metformin inhibits mitochondrial dysfunction and apoptosis induced by high glucose in cardiomyocytes through upregulation of AMPK activity [118]. Moreover, metformin protects against retinal ischemia/reperfusion injury through AMPK-mediated mitochondrial fusion (reversing the alteration in Mfn2 and OPA1 levels) [119].

Other substances, such as imeglimin (new oral agent for the treatment of T2D), NAD^+^ precursors, spermidine, urolithin A, and mitoquinone have shown positive effects on mitochondrial quality control and reduction in oxidative stress [120,121].

Interestingly, a recent case study described a NASH patient carrying a heterozygous MUL1 mutation, which led to the accumulation of dysfunctional mitochondria; this condition was mitigated by SGLT2 inhibitor treatment (50 mg of ipragliflozin) by activating residual MUL1 activity in this haploinsufficiency [122].

Further development of the “omic” techniques (e.g., genomics, proteomics) can evidence biomarkers that mediate these biological processes and key pathways related to alterations in the mitochondrial dynamics in a patient-personalized way, opening in the next future new perspectives for innovative diagnostic and targeted therapeutic solutions aimed to improve management of T2D and its complications. Accordingly, a proteomic approach evaluated 1250 circulating levels of intracellular proteins, measured at baseline, and following short and long-term treatment with empagliflozin in 1134 heart failure patients, was able to identify, among others, changes in the expression of proteins related to the enhancement of mitochondrial health and energy, repair, and regenerative capacity [123].

### 8.3. Epigenetic Modifications in T2D

Chronic activation of pro-inflammatory pathways in insulin target tissues (liver, muscle, adipose tissue and pancreatic islets), under the joint regulation of environmental factors and genetics combined with a variety of epigenetic modifications could contribute to T2D development [124] that is a typical metabolic inflammatory disease [125]. Epigenetic changes regulate the expression of many genes, including inflammatory ones [126,127,128,129,130] and several studies have found hundreds of epigenetic alterations in relation T2D in human tissues that are relevant to metabolism [131]. Epigenetic modifications have an important role in the development and progression of cardiovascular disease and T2D, in particular through DNA methylation, non-coding RNA regulation, and histone modifications that regulate the expression of genes implicated in oxidative stress, angiogenesis and inflammation [132,133]. Instead, few studies have paid attention to the ability of antidiabetic drugs and their effects on DNA methylation in terms of protective role at cardiovascular level. In human ventricular cardiac myoblasts exposed to high glucose for 1 week, the effects of empagliflozin (EMPA) on hyperglycemia-induced DNA methylation of NF-κB, SOD2, and IL-6 genes by pyrosequencing-based methylation analysis were investigated [134]. In this study, after seven days, human cardiomyocyte cell lines exposed to hyperglycemic conditions (HG) revealed significant oxidative stress evaluated by ROS concentration compared to normal glucose condition (NG), an effect counteracted by co-treatment with EMPA. Moreover, HG induces an increment in NF-κB, SOD2, and IL-6 mRNA expression compared to the NG condition, while the co-treatment with EMPA leads to a reduction in these markers of inflammation and oxidative stress. The same modulation was observed for protein concentration. HG treatment induces demethylation in the promoter regions of NF-kB and SOD2 which leads to an increase in mRNA expression of these genes. Co-treatment with EMPA reduced the expression levels and TET2 binding to the promoter region of NF-kB and SOD2, preventing the HG-induced demethylation and restoring the normal levels of gene expression [134]. Through metabolic analysis, Nishitani et al. showed higher levels of several intermediate metabolites of the glycolytic pathway and tricarboxylic acid (TCA) cycle in diabetic mice compared to normal mice; after dapagliflozin, the accumulation of glycolytic intermediate metabolites improved, although no significant effects were observed for the intermediate metabolites of the TCA cycle. Moreover, dapagliflozin increased plasma and adipose 3-hydroxybutyric acid (3-HBA) concentration. Microarray analysis showed that adipocytokines were downregulated in diabetic mice compared with non-diabetic mice but upregulated after dapagliflozin. In vitro 3-HBA induced β-hydroxybutyrylation of histone H3 at lysine 9 and upregulation of adiponectin in 3T3-L1 adipocytes independently of their acetylation or methylation, suggesting that 3-HBA could provide protection through epigenetic modifications of the adiponectin gene in adipocytes likely through direct modification of histone H3K9; this result may help to identify target genes of 3-HBA, in order to better understand the molecular mechanisms of SGLT2 inhibitors involved in the protection against cardiovascular events [135].

### 8.4. Gut Microbiota Profile in T2D

The gut microbiota, comprising microorganisms such as bacteria, yeasts, and viruses localized in the gastrointestinal tract, is characterized by a dynamic composition [136]. The adult intestine harbors a vast array of bacterial species, with an estimated number of bacterial classes ranging from 500 to 1000; compared to human cells, the number of bacterial cells in the intestinal microbiome exceeds that of the human body by tenfold [137]. Furthermore, the intestinal microbiome contains a significantly greater number of genes compared to the human genome, with over 150 times more genes [138]. Various studies have extensively examined the role of the gut microbiota in T2D, highlighting their close relationship [139]. In fact, intestinal dysbiosis, characterized by an imbalance in the microbiome composition and function, appears closely linked to obesity-induced metabolic disorders, including T2D [140,141]. Dietary habits and lifestyle significantly influence the complexity of the intestinal microbial ecosystem, regulating vital functions including immune responses and nutrient metabolism [142]. Factors like obesity, visceral fat accumulation, and medication use can contribute to dysbiosis. Therefore, characterizing the intestinal microbiota and identifying metagenomic biomarkers are crucial for diagnosing and managing T2D [143]. The intestinal microbiota is primarily composed of four key bacterial phyla: Proteobacteria, Firmicutes, Actinobacteria, and Bacteroidetes. Notably, specific intestinal bacteria have been identified as biomarkers associated with T2D and numerous recent studies have demonstrated a close correlation between the bacterial composition of the intestinal microbiota and the onset of T2D [144]. Specifically, analysis of the intestinal microbiota has revealed a decrease in bacterial diversity in T2D patients compared to healthy individuals, with a significant reduction in butyrate-producing bacteria such as Bifidobacterium and Akkermansia. These bacteria, known for their role in short-chain fatty acid production, have demonstrated beneficial effects on metabolic health, including blood glucose regulation and inflammation reduction [145] Conversely, some opportunistic pathogenic bacteria, such as Fusobacterium, Ruminococcus, and Blautia, have shown a positive association with T2D pathophysiology. These bacteria have been implicated in chronic inflammation and alterations in lipid and carbohydrate metabolism, contributing to T2D progression [146]. To date, specific genetic markers and gene clusters have been used to classify individuals with T2D. Karlsson et al. found that metagenomic profiles could be used to identify T2D with high accuracy, although further research is needed to develop metagenomic predictive tools and diagnostic biomarkers for specific populations [147]. Moreover, 16S rRNA sequencing could be a more convenient method for characterizing the microbiota [148]. Functional analysis of the intestinal microbiome has revealed significant correlations between increased levels of butyrate production through transferases, degradation of various amino acids, and T2D, as demonstrated by Li et al. [139]. Additionally, Karlsson et al. highlighted that elevated levels of butyrate production through specific bacterial metabolic pathways were associated with insulin resistance in T2D patients [147]. Furthermore, the intestinal microbiota involvement in the development of T2D via the metabolism of amino acids like tyrosine and alanine has been reported [139]. These findings have paved the way for novel therapeutic strategies targeting the intestinal microbiota, such as the use of probiotics, living microorganisms that provide health benefits, prebiotics, non-digestible substances that nourish beneficial bacteria in the colon, and dietary modifications, which could represent promising approaches for the prevention and treatment of T2D [149].

In the context of T2D drug therapy, attention is increasingly focusing on the complex interaction between antidiabetic drugs and the gut microbiota. This interaction is bidirectional: on one hand, these drugs can alter the composition of the intestinal microbiome by increasing the abundance of short-chain fatty acid (SCFA)-producing bacteria; on the other hand, the microbiota and SCFA influence the efficacy of antidiabetic agents, affecting pharmacogenetics and drug availability [150]. Understanding this bidirectional interaction is crucial for identifying potential mechanisms to modulate the microbiota.

In the case of metformin, the drug can increase the abundance of bacteria such as Akkermansia and other SCFA producers, thereby improving glucose concentrations in the patient. GLP-1 receptor agonists, such as liraglutide, can influence the composition of the microbiota by increasing the abundance of certain bacteria such as Allobacum and Turicibacter. DPP-4 inhibitors, like sitagliptin, can also modulate the microbiota by reducing the abundance of Firmicutes and increasing that of Bacteroidetes [151]. This growing understanding of the interaction between antidiabetic drugs and gut microbiota holds promise for the development of personalized therapeutic approaches that harness the potential of microbiota modulation in the management of T2D [152]. In this context, oral microbiota may also have a role in T2D, as oral bacteria can translocate and modify the composition of gut microbiota, modulating immunity, oxidative stress and inflammatory responses, and being targets of intervention (e.g., application of *Lactobacillus* in the clinical practice) [153].

Surely, the parallel development of statistical methods capable of analyzing and integrating the complex host–microbiome crosstalk and the specific mechanisms by which this relationship is modulated, will be also critical to interpret all data obtainable towards the optimization of the T2D diagnosis and management in a personalized way [153].

## 9. Discussion and Conclusions

Diabetes, although not an infectious disease, has been designated an epidemic by the WHO due to its prevalence and the rapid increase in the number of cases worldwide. According to reports from organizations dealing with research on diabetes, T2D is a particular problem, in which every second patient is unaware of the disease. Quick diagnosis and proper control of blood glucose levels are crucial for appropriate therapy to avoid the complications it causes. New drugs are becoming increasingly important in antidiabetic therapy. Thus, recent years have seen tireless efforts in the optimization of diabetes management in parallel to the improvement of understanding of diabetes pathophysiology and the proposal and testing of a number of potential new therapeutic agents that allow a better and more personalized approach to T2D treatment. Currently, each optimal treatment plan must include the evaluation of the risk profile of the patient, especially at the cardiovascular and renal level. This is made possible also thanks to the availability of new compounds (e.g., GLP-1 receptor agonists, SGLT2 inhibitors), that significantly benefit the cardiovascular system, as evidenced by results obtained in preclinical and experimental results (e.g., anti-oxidative, anti-inflammatory, and anti-atherogenic effects exerted on endothelial, inflammatory, and vascular smooth muscle cells and platelets) as well as those reported in cardiovascular outcome trials that provide a strong rationale for their use as cardiovascular drugs.

However, despite these recent exciting successes in the number and availability of T2D pharmacological bullets, there is still potential to fill the gap by increasing effectiveness, optimizing molecules and dosing regimens, and identifying patient groups that may particularly benefit most from the use of these drugs.

Diabetes and cardiology guidelines already include GLP-1RA in view of evidence-based beneficial effects: to decrease cardiovascular risk in high-risk individuals presenting T2D, to obtain adequate glucose control, improve weight loss, and minimize the risk of hypoglycemic events, also in combination with other hypoglycemic drugs (e.g., metformin), and lifestyle modification (diet and exercise, that as main pillars of a holistic diabetes care plan should be always strenuously encouraged) [154,155].

Nonetheless, although recommended in clinical guidelines from cardiovascular societies, many cardiovascular patients are not actually treated with these drugs because of low cardiologists’ prescription [156]. Analysis of SGLT2is and GLP-1RAs prescriptions suggested that the use of both drug classes significantly increased during in the last years; however, cardiologists, who are more subjected to visit patients with both T2D and CVD, accounted for much less that 10% of prescriptions for these drugs in 2020 [157,158,159]. Beyond the fact that these new drugs are more expensive compared with older ones, clinical apathy and lack of confidence in prescribing drugs which are perceived as pertaining to the diabetes sector could be reasons for this gap between clinical evidence and clinical practice. Collaboration between different specialties (e.g., researcher and clinicians as well as diabetologists and scientific professional societies, with primary care practitioners and specialists, including cardiologists) and better patient information and education is a critical issue to overcome current limitations and fill the gap between the under-use of cardioprotective antidiabetic agents in clinical daily practice among high-risk T2D patients and the clear endorsement of international guidelines.

Other new drugs and future molecules which are of interest for T2D treatment are in development, as new oral GLP-1 receptor agonists such as danuglipron or orforglipron [160,161].

Moreover, dual glucose-dependent insulinotropic polypeptide (GIP) and glucagon-like peptide-1 (GLP-1) receptor agonist (tirzepatide is already approved for the treatment of T2D in 2022 by FDA and in 2023 by EMA) or triple agonists, glucagon-like peptide 1 (GLP-1), glucose-dependent insulinotropic polypeptide (GIP), and glucagon agonists (retatrutide-LY3437943) can become increasingly important in antidiabetic therapy in the near future [162,163].

In particular, tirzepatide has been evaluated in the multicenter multinational SURPASS clinical trials (SURPASS-1 through SURPASS-5; 5, 10, and 15 mg subcutaneous injections once weekly) [164]. Table 6 shows the results of SURPASS-1-5 for tirzepatide treatment in T2D [165,166,167,168,169].

Altogether, available data obtained evidenced safety and effectiveness of this drug for T2D patients and its utility to achieve better glycemic control, especially in patients requiring body weight reduction and low hypoglycemia risk [170].

Thus, the scenario is rapidly evolving, increasing the instruments in the fight against T2D, in order to improve the patient cardiometabolic profile together with glycemic control. Nonetheless, different aspects for each available drug should be adequately evaluated; for example, the use of oral drugs, which may provide a valid alternative to injectable ones to achieve treatment targets, being less demanding for the patient, or the accurate assessment between benefits against side effects (e.g., nausea, vomiting) when considering the use of each new molecule in the clinical practice.

Moreover, novel insights into the role of epigenetic cell senescence and gut microbiota and mitochondrial dynamics make possible the development of new disease-modifying therapies that are able to bring new light and completely modify the frontiers of cardiometabolic management. However, the application of these innovations, although able to change the lives of millions of T2D people in the future, surely will require concerted and coordinated efforts by different professionals as well as a removal of traditional barriers among biological and medical specialties that likely will represent one of the main challenges in the medicine field of this century.

## Figures and Tables

**Table 1:** The “ominous octet” in T2D pathophysiology.

1. reduced insulin secretion by β-cells (pancreas)
2. increased glucagon secretion by ⲁ-cells (pancreas)
3.increased glucose production (liver)
4. increase lipolysis (adipose tissue)
5. reduced appetite (brain)
6. decreased incretin effect (bowel)
7. increased glucose (kidney)
8. decrease in glucose uptake (muscle)

**Table 2:** Main currently available GLP-1 RAs.

GLP-1RA	Frequency of Administration	Structural Class	Administration Route	Delivery	Dose	Contraindications	Adverse Effects
Liraglutide	Once/day	GLP-1	SCI	Multidose pen	0.6–1.8 mg	History of pancreatitis and pancreatitis development while taking these medications. Severe gastrointestinal diseases, hypersensitivity and pregnancy. Personal or family history significant for multiple endocrine neoplasia 2A, multiple endocrine neoplasia 2B, or medullary thyroid cancer	Nausea, vomiting, diarrhea; dizziness, mild tachycardia, infections, headaches, and dyspepsia may also occur. Low risk of minor hypoglycemic episodes. Combination with dipeptidyl peptidase-4 inhibitors not currently recommended due to not significant glycemic improvement but enhanced hypoglycemic effects. Still unclear possible interactions with other oral anti-diabetic medications.
Exenatide immediate release	twice/day	Exendin-4	SCI	Multidose pen	5–10 µg		
Exenatide long acting	once/week	Exendin-4	SCI	Single dose/powder and diluent supplied	2 mg		
Dulaglutide	once/week	GLP-1	SCI	Multidose pen/syringe	0.75–1.5 mg		
Semaglutide	once/week	GLP-1	SCI	Multidose pen	2.25–1 mg		
Oral semaglutide	once/day	GLP-1	O	Capsule	3–14 mg		
Lixisenatide	once/day	Exendin-4	SCI	Multidose pen	10–20 µg		

GLP-1 = modified human GLP-1; Exendin-4 = exendin-4 derivative; oral = O; subcutaneous injection = SCI.

**Table 3:** Advantages for glycemic control and cardiovascular benefits proposed for GLP-1RAs.

improved glucose control
minimal hypoglycemic events
improved endothelial function
body weight reduction
reduced blood pressure
improved lipid profile; reduced triglycerides
improved recovery after ischemia
a reduction in MACE events and CV mortality in high CV risk T2D patients
improved hemodynamics in patients with left ventricular dysfunction or heart failure

**Table 4:** Advantages for glycemic control and cardiovascular benefits proposed for SGLT2i.

improved glucose control
natriuretic effect (reduced risk of hospitalizations for heart failure and the progression of the renal damage)
minimal hypoglycemic events
improved endothelial function
body weight reduction
cardioprotection
reduced blood pressure
improved lipid profile
improvement of inflammation and oxidative stress
possible use for transthyretin amyloid cardiomyopathy
a reduction in MACE events and cardiovascular mortality in high cardiovascular risk T2D patients
a reduction in infarct size and the occurrence of ischemia-reperfusion-induced arrhythmias
reduction in heart failure risk

**Table 5:** Main currently available SGLT2 inhibitors.

SGLT 2 I	Frequency of Administration	Structural Class	Administration Route	Dose	Contraindications	Adverse Effects
Empagliflozin	Once/day	SGLT2 inhibitor	oral	10 mg and 25 mg	Patients with Type 1 diabetes; Pregnant women and breastfeeding, consequently; Impairment of renal glomerular filtration below 20 mL/min/1.73 m^2^; Symptoms of diabetic ketoacidosis	Increased risk of non-sexually transmitted genital infections and/or urinary infections (especially in women); Increased risk of plasma volume depletion and hypotension (especially elderly patients with moderate renal insufficiency or on therapy with loop diuretics); diabetic ketoacidosis (rare);
Dapagliflozin	Once/day	SGLT2 inhibitor	oral	10 mg		
Canagliflozin	Once/day	SGLT2 inhibitor	oral	100 mg and 300 mg		
Ertugliflozin	Once/day	SGLT2 inhibitor	oral	5 mg and 15 mg		

**Table 6:** Results of SURPASS-1-5 for tirzepatide treatment in T2D.

Trial Name	Trial Type	Trial Target	Trial Primary Endpoint	Trial Results	Reference
Surpass-1	randomized, double-blind, parallel-group, phase 3 trial	Effectiveness of tirzepatide (5, 10 or 15 mg; subcutaneous injections once weekly) compared to placebo in patients with T2D inadequately controlled by diet and exercise	Mean change in HbA1c from baseline at 40 weeks	Effectiveness of tirzepatide compared with placebo in glycemic control and weight loss, without increased risk of hypoglycaemia; safety profile comparable with GLP-1 receptor agonists	[165]
Surpass-2	randomized, parallel-group, open-label, phase 3 trial	Comparison of tirzepatide with the GLP-1R agonist Semaglutide	Mean change in HbA1c from baseline at 40 weeks	Tirzepatide at all doses noninferior and superior to semaglutide; loss weight greater with tirzepatide than with semaglutide. Most common adverse events were gastrointestinal (nausea, diarrhea, vomiting).	[166]
Surpass-3	randomized, open-label, parallel-group, phase 3 trial	Comparison of tirzepatide (once-weekly) with insulin degludec (once-daily) as an add-on to metformin with or without SGLT2 inhibitors	Mean change from baseline in HbA1c at week 52.	Tirzepatide at all doses superior to titrated insulin degludec, with greater reductions in HbA1c and bodyweight (week 52) and lower hypoglycaemic risk; safety profile comparable with GLP-1 receptor agonists	[167]
Surpass-4	randomized, open-label, parallel-group, phase 3 trial	Comparison of tirzepatide (10 or 15 mg, or both), with insulin glargine in patients with a high cardiovascular risk, inadequately controlled by oral glucose-lowering drugs	HbA1c change from baseline to 52 weeks.	Superior HbA1c reduction with a lower incidence of hypoglycaemia at week 52. Tirzepatide was not associated with excess cardiovascular risk.	[168]
Surpass-5	*randomized*, double-blind, phase 3 trial	Addition of subcutaneous tirzepatide, compared with placebo, to titrated insulin glargine in T2D patients inadequately controlled with basal insulin, with or without metformin.	HbA1c change from baseline to 40 weeks.	Improved glycemic control in terms of HbA1c reduction at week 40, with higher % of patients treated with tirzepatide vs those treated with placebo who showed HbA1c less than 7%; more weight reduction.	[169]
